# Age‐dependent DNA methylation patterns on the Y chromosome in elderly males

**DOI:** 10.1111/acel.12907

**Published:** 2019-02-21

**Authors:** Jesper B. Lund, Shuxia Li, Kaare Christensen, Jonas Mengel‐From, Mette Soerensen, Riccardo E. Marioni, John Starr, Alison Pattie, Ian J. Deary, Jan Baumbach, Qihua Tan

**Affiliations:** ^1^ Epidemiology and Biostatistics, Department of Public Health University of Southern Denmark Odense Denmark; ^2^ Unit of Human Genetics, Department of Clinical Research University of Southern Denmark Odense Denmark; ^3^ Centre for Genomic and Experimental Medicine University of Edinburgh Edinburgh UK; ^4^ Centre for Cognitive Aging and Cognitive Epidemiology University of Edinburgh Edinburgh UK; ^5^ Alzheimer Scotland Dementia Research Centre University of Edinburgh Edinburgh UK; ^6^ Department of Psychology University of Edinburgh Edinburgh UK; ^7^ Chair of Experimental Bioinformatics, TUM School of Life Sciences Weihenstephan Technical University of Munich Munich Germany

**Keywords:** age‐dependent patterns, aging, DNA methylation, mortality, Y chromosome

## Abstract

The Y chromosome, a sex chromosome that only exists in males, has been ignored in traditional epigenetic association studies for multiple reasons. However, sex differences in aging‐related phenotypes and mortality could suggest a critical role of the sex chromosomes in the aging process. We obtained blood‐based DNA methylation data on the Y chromosome for 624 men from four cohorts and performed a chromosome‐wide epigenetic association analysis to detect Y‐linked CpGs differentially methylated over age and cross‐validated the significant CpGs in the four cohorts. We identified 40–219 significant CpG sites (false discovery rate <0.05) with >82% of them hypermethylated with increasing age, which is in strong contrast to the patterns reported on the autosomal chromosomes. Comparing the rate of change in the Y‐linked DNA methylation across cohorts that represent different age intervals revealed a trend of acceleration in DNA methylation with increasing age. The age‐dependent DNA methylation patterns on the Y chromosome were further examined for their association with all‐cause mortality with results suggesting that the predominant pattern of age‐related hypermethylation on the Y chromosome is associated with reduced risk of death.

## 1 INTRODUCTION

The Y chromosome, the sex‐determining chromosome found only in the male phenotype of the population, contains circa 57 million DNA base pairs (BP; Million BP: MP; H. Sapiens build: hg38/GRCh38) and is often neglected in (epi)genetic studies. With its “small” size, it is only larger in terms of BP than both chromosome 21 (*circa *47 MP) and chromosome 22 (*circa *51 MP). The Y chromosome used to be considered mostly meaningless with a high percentage of repetitive and noncoding regions (sometimes referred to as “genomic deserts”). With improvements in analytical methods and technologies (Jobling & Tyler‐Smith, 2003, 2017), the chromosome has become a more popular analysis target. The Y chromosome is most commonly studied regarding Y‐linked haplogroups (Consortium YC, 2002; Knijff, 2000; Zerjal et al., 1997), especially within phylogeny due to its nature of low recombination and paternal inheritance. Moreover, loss of Y chromosome (LOY) has been observed with age (Forsberg, 2017) and Y‐chromosomic deletions on specific regions (Yq11) that are associated with oligozoospermia and azoospermia phenotypes can cause infertility of different degrees (Vog et al., 1996).

In comparison with the recent upsurge in omics studies focusing on autosomal chromosomes, the sex chromosomes are often not included in analyses, especially within genome‐wide association studies (GWAS), epigenome‐wide association studies (EWAS), and transcriptome‐wide association studies (TWAS) on complex diseases and traits (Wise, Gyi, & Manolio, 2013). This is unfortunate because the sex chromosomes could be influential (directly or indirectly) on certain diseases with sex differences (Khramtsova et al. 2019). Particularly, in the field of aging research, sex differences have been found to affect the trajectory of aging phenotypes (Dowling, 2014), aging‐related diseases such as Alzheimer disease and other dementias (Mazure & Swendsen, 2016), and mortality (Austad & Fischer, 2016; Case & Paxson, 2005). Although multiple EWASs have been performed to study the dynamic regulatory patterns of the aging methylome, current literature concerning associations between the Y chromosome and aging mainly describes LOY and copy number variants (CNV) (Zhou et al., 2016), which have been reported as far back as 1972 (Pierre & Hoagland, 1972).

Making use of existing multiple datasets on genome‐wide DNA methylation in older male subjects, we performed an exploratory Y chromosome‐wide association study on the aging‐related methylation changes on the Y chromosome and compared them with those from the autosomal chromosomes. We replicate findings across datasets and correlate age‐related methylation changes with all‐cause mortality and discuss potential implications in the epigenetics of aging.

## 2 RESULTS

### 2.1 Age patterns of methylation in Y‐linked CpGs

From the four datasets MADT, LASDT1, LSADT2, and LBC1921, we identified 219, 76, 40, and 169 CpGs displaying age‐dependent methylation patterns with FDR<0.05, respectively. Among them, 207, 72, 35, and 138 CpGs were hypermethylated over age, accounting for 95%, 95%, 88%, and 82% of all significant CpGs in each cohort. There were 12, 4, 5, and 31 CpGs hypomethylated with age, representing 5%, 5%, 12%, and 18% of all significant CpGs found in each cohort. The results show a high percentage of hypermethylated CpGs with age on the Y chromosome.

### 2.2 Cross‐sample/population replication of significant CpGs

An overall view of the above four sets of CpGs revealed a total of 282 CpGs significantly hypermethylated with FDR <0.05 in at least one cohort (Supporting Information Table S1), where 139 of the sites were also significantly hypermethylated in at least one other cohort with FDR <0.05. For the hypomethylated sites, 48 were significant in at least one of the cohorts with FDR <0.05 (Supporting Information Table S2) but with little validation (*N* = 3) in any of the other cohorts with FDR <0.05.

Table 2 presents, for each dataset in the discovery column, the proportion of significant CpGs replicated across the other three datasets. In general, hypermethylated CpGs were much more replicated in comparison with hypomethylated CpGs. The 72 and 35 hypermethylated CpGs detected by LSADT2 and LSADT1 had a replication rate of over 90% by the MADT cohort. Even the 138 hypermethylated CpGs discovered by the Scottish LBC1921 birth cohort were replicated with 43% by the Danish MADT cohort. In the rightmost column of Table 2, we show the proportion of discovery CpGs replicated by any of the three other datasets. It shows again, the hypermethylated CpGs were much more replicated than the hypomethylated CpGs. In Table 3, we show detailed information for 7 CpGs that are present in all four cohorts with FDR <0.05. All of them are hypermethylated with increasing age. Among the 7 CpGs, 5 are linked to functional genes or pseudogenes at the promoter regions.

### 2.3 Accelerated rate of hypermethylation in high age‐groups

**Figure 1:**
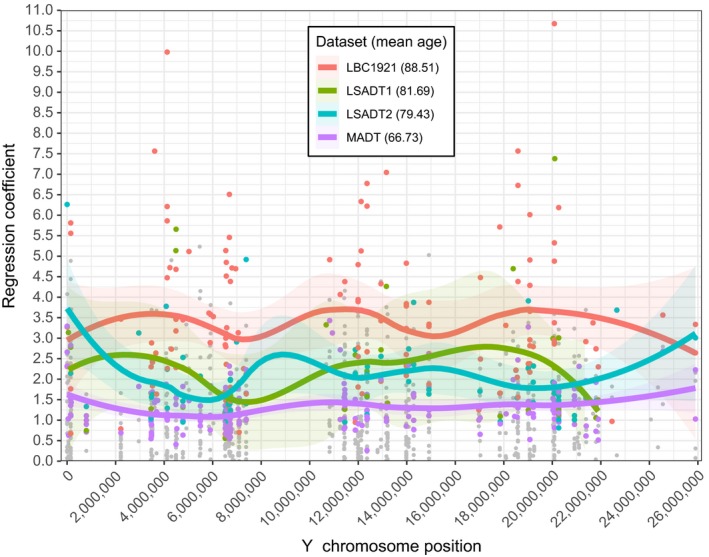
Plotted coefficients of the discovery analysis over their chromosome position. Trend lines were fitted by local polynomial regression fitting (LOESS). Colored dots are significant sites (FDR <0.05). Gray dots are nonsignificant sites. Mean cohort age: 66.73, 79.43, 81.69, and 88.51 for MADT (purple), LSADT2 (blue), LSADT1 (green), and LBC1921 (red), respectively

By plotting the coefficients of the significant hypermethylated CpGs over Y chromosome position (Figure 1), we see a tendency of increased hypermethylation profiles with increased age. We smoothed the regression coefficients for age (the rate of change) using locally weighted scatterplot smoother (Jacoby, 2000) (LOESS, *α* = 0.5), with residual standard errors of 0.51, 1.57, 0.917, and 1.772, for MADT, LSADT1, LSADT2, and LBC1921, respectively. These lines clearly demonstrate a trend of a higher magnitude of regression coefficients for hypermethylation for cohorts of higher mean ages. More specifically, LBC1921 (mean age: 88.51) has higher coefficient values compared with MADT (mean age: 66.73), LSADT1 (mean age: 79.34), and LSADT2 (mean age: 81.69). The lines for LSADT1 and LSADT2 are intertwined but still notably higher compared to the line of MADT.

**Figure 2:**
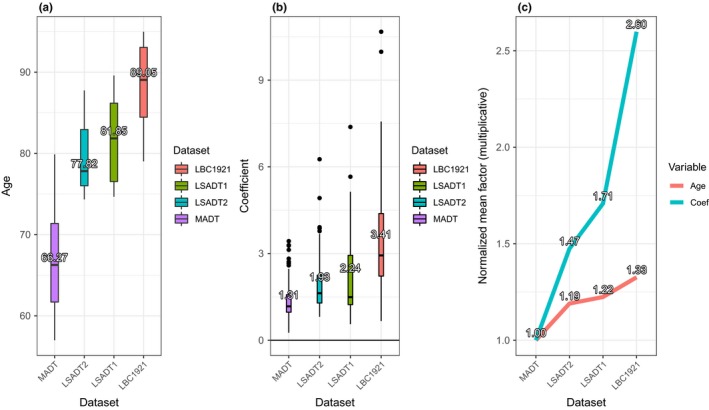
Plots displaying accelerated increase in DNA methylation by significantly hypermethylated CpGs (FDR <0.05) in the four cohorts ordered by increasing mean age. The boxplots in 2a and 2b show the distribution of sample ages and regression coefficients of hypermethylated CpGs in each cohort. Acceleration in DNA hypermethylation with increasing age is clearly illustrated by plotting, for each cohort, the means of ages and means of regression coefficients normalized by MADT (2c)

Figure 2 displays the box plots for cohort age (a) and for the regression coefficients of significantly hypermethylated CpGs by dataset (b). We see a tendency of higher age with higher methylation level. The tendency is seen when looking at Figure 2c. Here, we see that the mean increase in age of 133% (factor 1.33, mean age 66.73–88.51 years) corresponds to the accelerated hypermethylation values by 260% percent (mean coefficients of 1.31–3.40).

Among the significant CpGs (FDR <0.05), 7 were present in all four cohorts (Table 3 **)**. Again, we checked how the coefficient of these corresponded to findings above. We ranked the coefficient of each site from each cohort to numbers between 1 and 4, where 1 was the highest value and 4 was the lowest. We saw that for all but one sites, LBC1921 had the highest value (score: 1). For MADT, all sites had the smallest value (score: 4). For LSADT1 and LSADT2, the scores were all either 2 or 3, except for a single site having score 1. Again here, we are able to reveal how the higher ages in the cohorts correspond to higher coefficients for the 7 CpGs.

By performing the Wilcoxon rank‐sum test (also known as Mann–Whitney–Wilcoxon (MWW) or *U* test) on all sites where the coefficients in all cases were hypermethylated (*N* = 125), revealed the same conclusions as above. The 125 sites were picked out on the basis that only the sites that at least one of them were significantly associated with age (FDR<0.05) in at least one cohort but had positive regression coefficients (i.e., increased methylation over age) for all four datasets. With an H_0_ = no difference in coefficients between the cohorts (where the main difference of the cohorts is their age), the test results are produced in Table 4. For all tests except the comparison between LSADT1 and LSADT2 (*p* = 0.80), a significant difference was observed, with *p* = 2.7832e‐06 for comparing LBC1921 with MADT. We can conclude that there are significant differences in the coefficients (i.e., the rate of change) between older and younger cohorts, as suggested by Figures 1 and 2.

### 2.4 Relationship with mortality

**Figure 3:**
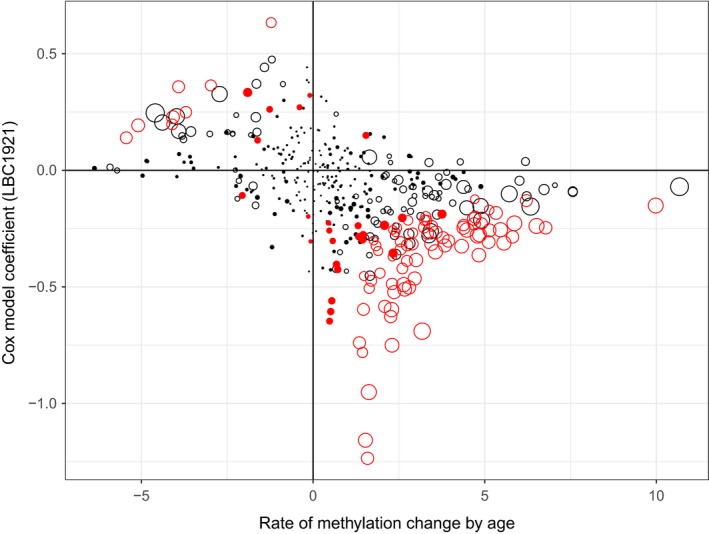
Scatterplot displaying relationship between age‐related rate of change in DNA methylation and mortality. The empty dots are CpGs significantly methylated with age (FDR <0.05, the larger the dots the higher the statistical significance). The red dots are CpGs associated with mortality with *p* < 0.05 in the Cox regression model. The figure shows that age‐associated CpGs predominantly contribute to reduced risk of death

The LBC1921 birth cohort contains samples of older ages with information on mortality available. This enables us to compare the association of age‐related CpGs on the risk of death. Among the 169 age‐associated CpGs, about half (74 CpGs, 44%) have *p* < 0.05 in the survival analysis using Cox regression (Supporting Information Table S3). In Figure 3, we display the coefficients from the Cox regression model against the coefficients on age (the rate of change) for methylation. CpGs significantly hyper‐ or hypomethylated with age (FDR<0.05 for empty dots; the larger the dots, the higher the significance level) also tend to be significantly associated with mortality (red dots *p* < 0.05 from Cox model). The relationship is dominated by CpGs hyper‐ or hypomethylated with age (large dots) that are associated with a lower hazard of death (negative coefficients from Cox model for hypermethylated CpGs in the bottom‐right panel while positive coefficients from Cox model for hypomethylated CpGs in the top‐left panel). The collective association with mortality (indicated by incremental *R* ^2^) by age‐associated CpGs is shown in Supporting Information Figure S1 where a PMS based on a list of top 30 CpGs best fits to the mortality data with a *p*‐value of 9.59e‐03 and adjusted *R* ^2^ of 0.035. Interestingly, the regression coefficient for all PMSs is negative (Supporting Information Table S4) indicating a negative correlation with mortality.

## 3 DISCUSSION

By focusing on male‐only samples, we were able to analyze the age pattern of Y‐linked DNA methylation in older people. We identified significant CpG sites that change their methylation levels across ages. Different from the reported age‐related methylation patterns dominated by decreased methylation over increasing ages (Johansson, Enroth, & Gyllensten, 2013; Li et al., 2017; Marttila et al., 2015), the Y‐linked DNA methylation is characterized by increased methylation with increasing age, accounting for over 80% or 90% of all the significant age‐associated CpGs. As shown by Figure 3, the CpGs hypermethylated with increasing age nearly all have negative coefficients from the Cox model indicating an association with longer survival by age‐related methylation changes. For the small group of CpGs with increased hazard of death when methylation goes up, their methylation levels are in fact decreased with increasing age meaning nonincreased or even decreased risk of death by their age‐related methylation patterns. This is further supported by the Cox model for PMS with a negative coefficient showing that increased score of multiple age‐related CpGs (mostly hypermethylated with age) reduces the risk of death. We postulate that the observed age‐associated hypermethylation on the Y chromosome could represent an active response to the aging process that helps to maintain male survival at high ages.

The detected age‐associated hypermethylation on the Y chromosome is further consolidated by our cross‐cohort replication as shown in Table 2. High replication rates were observed across the datasets for hypermethylated CpGs while that for the hypomethylated CpGs were mostly low. This contrast suggests that the observed overwhelming pattern of Y‐linked hypermethylation could represent a striking difference in the age‐related epigenetic control over sex and autosomal chromosomes as the age‐associated methylation patterns on autosomal chromosomes are dominated by reduced methylation with increasing age (Li et al., 2017). We have recently compared the age‐associated CpGs with mortality‐associated CpGs on autosomal chromosomes found in the LBC1921 birth cohort and found very limited overlap (about 10% of the age‐associated CpGs) between them although the overlap is significantly different from random (unpublished results). Most importantly, the overlapping CpGs are dominated by those age‐related methylation patterns help to reduce mortality. Different from the autosomal chromosomes, the high overlap (44%) between age‐ and mortality‐associated CpGs on the Y chromosome (Figure 3) highlights its high importance in successful aging in males.

Among the 5 annotated genes in Table 3, increased expression of NLGN4Y has been associated with autism (Ross, Tartaglia, Merry, Dalva, & Zinn, 2015) and expression of DDX3Y may modulate neuronal differentiation (Vakilian et al., 2015). A recent study reported that the expression of the TBL1Y gene plays an important role in cardiac differentiation (Meyfour et al., 2017). The other gene in Table 3 (TTTY23, TTTY20, LOC100101121, or TTTY23B) is all nonprotein coding genes involved perhaps in the regulatory domain. Although current literature on these genes is limited and may not be directly linked with aging and mortality, their biological roles merit further investigations.

The Y chromosome accounts for about 2% of the total length of human genetic materials. However, the number of Y‐linked CpGs on the Illumina 450 K array (416 CpGs) is less than 0.1% of the total number of CpGs on the array (485,242 CpGs). This means that the CpGs for the Y chromosome are highly underrepresented. This can be due to the fact that the Y chromosome is a gene‐poor area (75–80 genes), in comparison with the number of genes carried by chromosome 20 (500–600 genes) which is about the same size. Comparing the number of CpGs on Y chromosome and chromosome 20 (10,383 CpGs), the proportion of Y‐linked CpGs is very much limited. This presents an obvious limitation of our study, and as such, our results should be interpreted with caution. We hope that future studies using high capacity design or methylation sequencing technique will help to validate our findings and uncover the impact of Y chromosome on male aging.

## 4 MATERIALS AND METHODS

### 4.1 Study populations and samples

We analyzed Y chromosome data on four cohorts of middle‐ and older‐aged subjects consisting of Middle‐Aged Danish Twins (MADT) (Gaist et al., 2000), Longitudinal Study of Aging Danish Twins (LSADT1, LSADT2) (Christensen, Holm, Mcgue, Corder, & Vaupel, 1999), and Lothian Birth Cohort of 1921 (LBC1921) (Deary, Gow, Pattie, & Starr, 2011). All four cohorts utilized DNA isolated from whole‐blood samples from individuals between the age of 56 and 95 which have been processed using the Illumina HumanMethylation450 BeadChip or 450 K array (Illumina, Inc., San Diego, CA, USA). For this study, only male participants (*N* = 624), probes corresponding to the CpGs located on the Y chromosome (*N* = 416), and the latest wave sample of each participant from LBC1921 were used. Table 1 outlines the basic cohort characteristics.

**Table 1:** Cohort‐specific characteristics ordered by mean age

	MADT	LSADT2	LSADT1	LBC1921	Total
*N* (males)	266	72	48	238	624
Mean age (year) ± *SD*	66.73 ± 6.18	79.43 ± 4.08	81.69 ± 5.28	88.51 ± 4.74	77.66 ± 11.2
Age min/max (year)	56.99–79.87	74.33–87.75	74.66–89.59	79.01–94.97	56.99–94.97
Country of origin	Denmark	Denmark	Denmark	Scotland	

**Table 2:** Cross‐cohort replication for each set of hyper‐ and hypomethylated CpGs. Rows indicate discovery cohorts (*N*: the number of significant CpGs with FDR <0.05), and column indicate replication cohorts with percentages in the table showing the replication rate). The last column is proportion of discovery CpGs replicated by at least one replication cohort

	Replication				
Discovery	MADT	LSADT2	LSADT1	LBC1921	Any
Hypermethylated					
MADT (*N* = 207)		31.40%	15.46%	28.50%	57.00%
LSADT2 (*N* = 72)	90.28%		30.56%	27.78%	95.83%
LSADT1 (*N* = 35)	91.43%	62.86%		8.57%	94.29%
LBC1921 (*N* = 138)	42.75%	14.49%	2.17%		46.38%
Hypomethylated					
MADT (*N* = 12)		0.00%	8.33%	8.33%	16.67%
LSADT2 (*N* = 4)	0.00%		0.00%	25.00%	25.00%
LSADT1 (*N* = 5)	20.00%	0.00%		0.00%	20.00%
LBC1921 (*N* = 31)	3.23%	3.23%	0.00%		6.45%

**Table 3:** The 7 CpGs significantly hypermethylated in all four datasets (FDR <0.05)

Cross‐cohort hypermethylated CpGs (*N* = 7, FDR <0.05)					Illumina 450 K annotation^a^		
CpG	MADT_coef_ b	LSADT2_coef_ b	LSADT1_coef_ b	LBC1921_coef_ b	Gene (Name)	Gene (Group)	CpG Island
cg03055837	0.897143 (4)	1.008964 (3)	1.399633 (2)	2.602283 (1)	NLGN4Y	TSS1500	N_Shore
cg00311963	0.89143 (4)	0.988388 (3)	1.17261 (2)	1.917789 (1)	LOC100101121;TTTY23	TSS1500	S_Shore
cg00679624	0.836824 (4)	1.03516 (3)	1.069887 (2)	2.545285 (1)			Island
cg14180491	1.133525 (4)	2.143094 (2)	2.780263 (1)	2.068691 (3)	DDX3Y	5'UTR;1stExon	Island
cg01707559	1.194551 (4)	1.733023 (2)	1.228877 (3)	4.47433 (1)	TBL1Y	TSS200	Island
cg18188392	1.989856 (4)	2.556912 (3)	2.715966 (2)	3.89269 (1)			Island
cg06636270	1.017389 (4)	2.124417 (2)	1.581355 (3)	2.837249 (1)	TTTY20	TSS1500	N_Shore

a UCSC annotation, University of California, Santa Cruz.

b The coefficients are based on regression coefficients for age, and the number in parenthesis indicates order ranked from highest to lowest coefficient (1–4).

**Table 4:** Wilcoxon rank‐sum test *p*‐values on 125 CpGs hypermethylated in all four cohorts with FDR <0.05 in at least one cohort

	MADT	LSADT2	LSADT1	LBC1921
MADT		0.0103	0.0220	2.7838e−06
LSADT2	0.0103		**0.7997**	0.0052
LSADT1	0.0220	**0.7997**		0.0034
LBC1921	2.7832e−06	0.0052	0.0034	

The bold indicates nonsignificant differences in the coefficients of compared significantly hypermethylated CpGs.

### 4.2 Preprocessing and quality control (QC)

Before statistical analysis, the three Danish cohorts (MADT, LSADT1, and LSADT2) were normalized internally only by their Y‐linked CpGs (*N* = 416). This was done, using the *minfi* R package (Aryee et al., 2014) through subset‐quantile within array normalization (SWAN) (Maksimovic, Gordon, & Oshlack, 2012). The Scottish (LBC1921) cohort was already normalized using this package, but for the whole genome and was additionally adjusted for a minor batch effect revealed by principal component analysis (PCA) using the *ComBat* (Johnson, Li, & Rabinovic, 2007) function from the *sva* R package (Leek, Johnson, Parker, Jaffe, & Storey, 2012). Subsequently, we performed QC by removing probes with an overall sum of 10 or more cross‐reactive targets (*N* = 18) (Y‐a et al., 2013). No probes with detection *p *> 0.01, no‐signaling, polymorphic probes with European allele frequency at least 1% or more than 5% percent missing values were found. A total of 398 CpGs remained after preprocessing. For each CpG, methylation, β value was calculated as M/(M + U) with M and U for the methylated and unmethylated signal intensities. Before statistical modeling, methylation β values were converted into M values for better statistical properties by logit transformation.

### 4.3 CpG‐based association tests

The CpG‐based age association tests were modeled using linear regression models. For the Danish twin samples, twin pairing was included as a random factor in a mixed effect model. The regression analysis adjusted for blood cell‐type composition (CD8T, CD4T, natural killer cell (NK), B cell, monocyte, and granulocyte) estimated using Houseman's method (Houseman et al., 2012) implemented in the R package *minfi* for the Danish twin data and R package *celltypes450 *for the LBC1921 data.

The model for the twin cohorts was defined as:

$$\begin{array}{c} {\text{DNAm}}_{\text{CpG}}\;=\;\beta_{0}\;+\;\beta_{1}\text{age}\;+\;\beta_{2}\text{CD8T}\;+\;\beta_{3}\text{CD4T}\\ +\;\beta_{4}\text{NK}\;+\;\beta_{5}\text{Bcell}+\;\beta_{6}\text{Mono}\\ +\;\beta_{7}\text{Gram}\;+\;1|{\text{TwinPair}}_{\text{ID}} \end{array}$$

while for LBC1921, the model was defined as:

$$\begin{array}{c} {\text{DNAm}}_{\text{CpG}}\;=\beta_{0}\;+\;\beta_{1}\text{age}\;+\;\beta_{2}\text{CD8T}\;+\;\beta_{3}\text{CD4T}\;+\;\beta_{4}\text{NK}\\ +\;\beta_{5}\text{Bcell}\;+\;\beta_{6}\text{Mono}\;+\;\beta_{7}\text{Gram} \end{array}$$

For both, DNAm is methylation level for a CpG, that is, the methylation M value. The coefficient $\beta_{1}$of these regression models captures the mean pattern of DNAm changes over age or the rate of change in DNA methylation by age. Multiple testing was adjusted by calculating the false discovery rate (FDR, Benjamani–Hochberg; Benjamini & Hochberg, 1995), and CpGs with FDR <0.05 were defined as significant.

Besides analyzing the age‐dependent methylation patterns of Y‐linked CpGs, we also perform survival analysis to estimate the effect of DNA methylation on the risk of death in the LBC1921 samples (*N* = 238, death = 151) using the Cox proportional hazard model,

$$h\left(t\right)\;=\;h_{0}\left(t\right)\exp\left(\beta_{1}\text{DNAm}\;+\;\beta_{2}\text{age}\right)$$

here, *t* is the survival time (from age at blood sampling to death or to last follow‐up if censored), *h*(*t*) is the hazard function, and *h*_0_(*t*) is the baseline hazard function. The effect of DNA methylation on survival is adjusted for age at blood sampling.

### 4.4 Polygenic methylation score (PMS)

To summarize the effect of age‐associated Y‐linked CpGs on mortality, we use PMS as introduced by Linnér et al. (2017). For a list of q age‐associated CpGs selected using a significance cutoff, the PMS for a sample j is calculated as the sum of their coefficients for age (β) multiplied by methylation levels of corresponding CpGs (DNAm), ${\text{PMS}}_{j}\;=\;\sum_{i=1}^{q}\beta (i)*\text{DNAm}(i,j)$. Effect on mortality for the calculated PMS is assessed by including it as a variable in the Cox regression model together with individual age as a covariate for adjustment. The predictive power of PMS is evaluated by the incremental coefficient of determination (incremental *R* ^2^) calculated as the difference in *R* ^2^ (pseudo *R* ^2^) between the Cox model fitted with PMS and age and the model with age only.

## ETHICS APPROVAL

The MADT study was approved by the Regional Committees on Health Research Ethics for Southern Denmark (S‐VF‐19980072). The LASDT projects were approved by the Danish Scientific Ethics Committees. Written informed consents were obtained from all participants in the Danish twin studies. Ethics permission for the LBC1921 study protocol was obtained from the Multi‐Centre Research Ethics Committee for Scotland (MREC/01/0/56) and from Lothian Research Ethics Committee (LREC/2003/2/29).

## CONSENT FOR PUBLICATION

Written consent was obtained for all authors.

## CONFLICT OF INTEREST

None declared.
