# Supplementary figures and images for: Age‐dependent DNA methylation patterns on the Y chromosome in elderly males
Source: Aging Cell. 2019 Feb 21;19(2):e12907. doi: 10.1111/acel.12907 (PMC6996942; doi:10.1111/acel.12907)

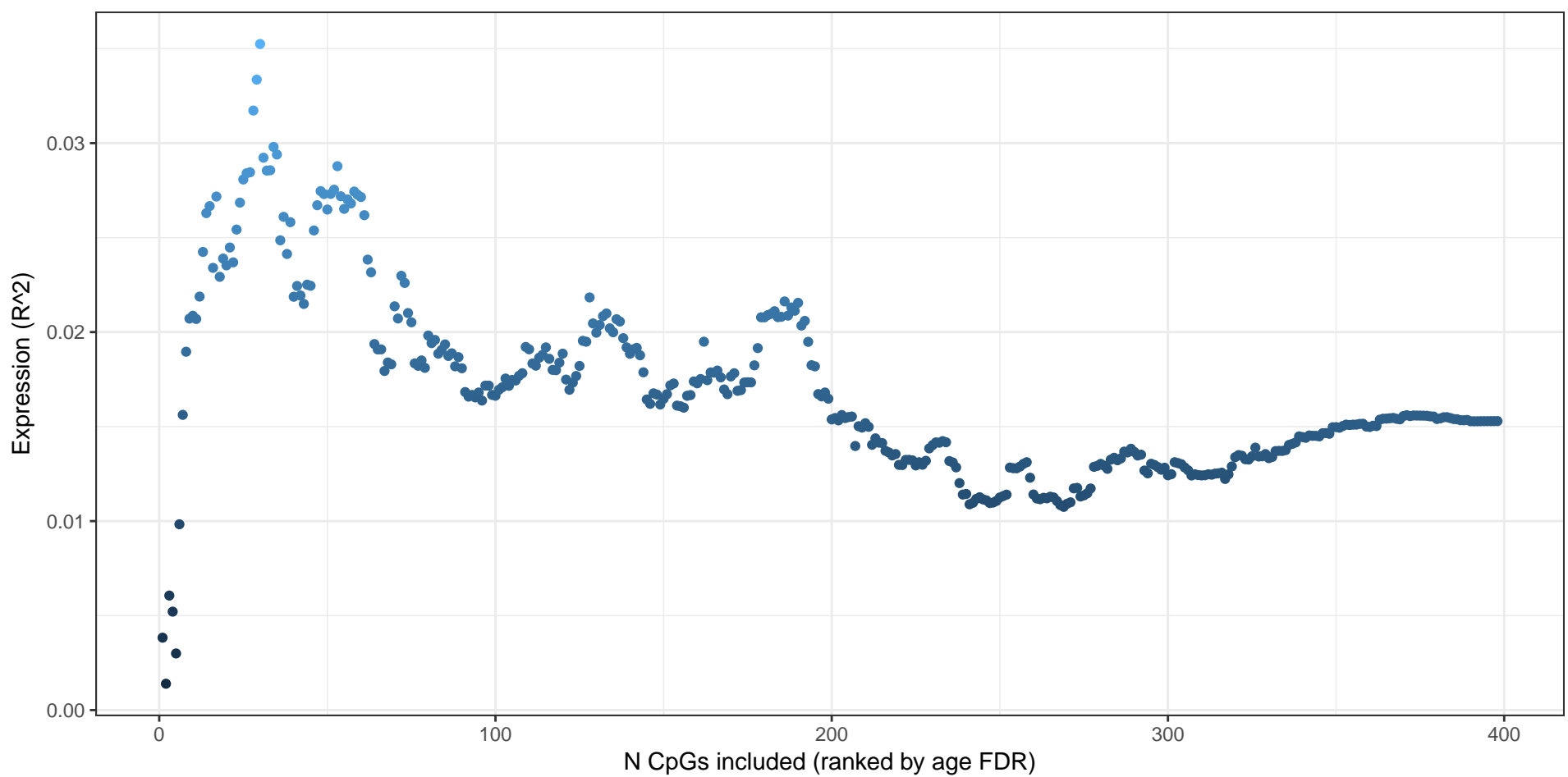

Supplement: Supplementary file 1 [file ACEL-19-e12907-s001.pdf]
